# Hepatic Gene Expression Profiles Differentiate Steatotic and Non-steatotic Grafts in Liver Transplant Recipients

**DOI:** 10.3389/fendo.2019.00270

**Published:** 2019-04-30

**Authors:** Ondrej Šeda, Monika Cahová, Irena Míková, Lucie Šedová, Helena Daňková, Marie Heczková, Miriam Brátová, Nikola Ďásková, Denisa Erhartová, Václav Čapek, Blanka Chylíková, Pavel Trunečka

**Affiliations:** ^1^First Faculty of Medicine, The General University Hospital, Institute of Biology and Medical Genetics, Charles University, Prague, Czechia; ^2^Centre for Experimental Medicine, Institute for Clinical and Experimental Medicine, Prague, Czechia; ^3^Department of Hepatogastroenterology, Institute for Clinical and Experimental Medicine, Prague, Czechia

**Keywords:** microarray, non-alcoholic fatty liver disease (NAFLD), transcriptomics profile, predictive signature, liver transplant

## Abstract

**Background:** Liver transplantation leads to non-alcoholic fatty liver disease or non-alcoholic steatohepatitis in up to 40% of graft recipients. The aim of our study was to assess transcriptomic profiles of liver grafts and to contrast the hepatic gene expression between the patients after transplantation with vs. without graft steatosis.

**Methods:** Total RNA was isolated from liver graft biopsies of 91 recipients. Clinical characteristics were compared between steatotic (*n* = 48) and control (*n* = 43) samples. Their transcriptomic profiles were assessed using Affymetrix HuGene 2.1 ST Array Strips processed in Affymetrix GeneAtlas. Data were analyzed using Partek Genomics Suite 6.6 and Ingenuity Pathway Analysis.

**Results:** The individuals with hepatic steatosis showed higher indices of obesity including weight, waist circumference or BMI but the two groups were comparable in measures of insulin sensitivity and cholesterol concentrations. We have identified 747 transcripts (326 upregulated and 421 downregulated in steatotic samples compared to controls) significantly differentially expressed between grafts with vs. those without steatosis. Among the most downregulated genes in steatotic samples were *P4HA1, IGF1*, or fetuin B while the most upregulated were *PLIN1* and *ME1*. Most influential upstream regulators included *HNF1A, RXRA*, and *FXR*. The metabolic pathways dysregulated in steatotic liver grafts comprised blood coagulation, bile acid synthesis and transport, cell redox homeostasis, lipid and cholesterol metabolism, epithelial adherence junction signaling, amino acid metabolism, AMPK and glucagon signaling, transmethylation reactions, and inflammation-related pathways. The derived mechanistic network underlying major transcriptome differences between steatotic samples and controls featured *PPARA* and *SERPINE1* as main nodes.

**Conclusions:** While there is a certain overlap between the results of the current study and published transcriptomic profiles of non-transplanted livers with steatosis, we have identified discrete characteristics of the non-alcoholic fatty liver disease in liver grafts potentially utilizable for the establishment of predictive signature.

## Introduction

Non-alcoholic fatty liver disease (NAFLD) is the most common chronic liver disease in industrialized countries, its prevalence being estimated at 19–31.3% (1). It encompasses a range of conditions that are thought to arise from fatty liver (simple steatosis) through non-alcoholic steatohepatitis (NASH), which refers to findings on liver biopsy reflecting typical metabolic and inflammatory changes (fat-related inflammation) with or without fibrosis in the absence of significant alcohol consumption (2, 3). NASH may develop into liver cirrhosis and hepatocellular carcinoma. Although NAFLD itself may be rather benign in the majority of patients, it is associated with increased specific (liver-related) mortality (4) and represents a substantial risk particularly for both fatal and non-fatal adverse cardiovascular events (5, 6) and chronic kidney disease (7).

The pathogenesis of NAFLD is multifactorial and multiple genetic and behavioral factors were identified to contribute to hepatic mishandling of fatty acids and carbohydrates as energy sources, as recently reviewed in detail. The overload of fatty acids in hepatocytes is deemed to result in the formation of lipotoxic species, paving the way toward NASH and fibrosis (5). As no targeted pharmacological therapy for NASH is currently in general clinical use, the modification of lifestyle is of particular importance in the prevention and therapy of this condition. Physical exercise, diet, and their combination belong to major modifiable aspects of non-pharmacological management of NAFLD. Reduction in caloric intake and macronutrients such as fat and carbohydrate have been utilized, sometimes with supplementation of natural antioxidants, with the aim to treat NAFLD (8–10). Although results of the studies have been so far somewhat conflicting, consistent effects leading to alleviation of NAFLD were repeatedly shown for weight loss and physical exercise (11).

It has been previously reported that among liver transplant recipients, post-transplant NAFLD affects 18–40% of subjects (12–14) and even 39–70% (15, 16) of those transplanted for NAFLD-related cirrhosis. At our Center, we performed a study based on repeated liver graft biopsies up to 15 years from liver transplantation and diagnosed graft steatosis in 56.4% patients, a proportion significantly exceeding the NAFLD prevalence in general population (17). In that study, liver graft steatosis was associated with a trend for diminished survival (mostly due to cardiovascular and oncologic reasons), and graft steatosis is considered a risk factor for poor outcome (18).

In clinical practice, simple steatosis is a frequent finding on ultrasonography, computer tomography or magnetic resonance examination. Nevertheless, the disease progression and impact on patient survival over time is the result of complex and dynamic interactions among many processes and the pathophysiological mechanisms leading to the development of a progressive and deleterious form of damage to the liver graft post-transplant as well as to the liver in general population are incompletely understood. The possibility to identify markers indicating the pro-steatotic condition of the graft prior to clinical manifestation of NAFLD may allow to start the specific therapy early and to reverse the negative trend. Moreover, we assume that the situation in liver graft recipients could serve as a valuable model for the study of NAFLD applicable to the general population. Liver transplantation offers a unique opportunity to follow graft recipients since engraftment, and unlike in general population, most factors putatively involved in pathogenesis could be extensively studied during the comprehensive follow-up including the repeated graft biopsies.

While the gene expression and other “-omic” signatures have been recently reported mostly for distinct oncological conditions including liver cancer (19), the systems biology-level analyses indicate that similar types of analyses applied to NAFLD might produce clinically-relevant results (20–22). Therefore, the aim of the presented study was to identify and contrast the gene expression profiles in liver graft biopsies in a cohort of patients either with or without histologically proven steatosis who successfully underwent liver transplantation.

## Materials and Methods

### Cohort

The cohort consisted of unselected adult liver transplant recipients who received cadaveric whole or partial liver graft in between 1995 and 2013 at the Institute for Clinical and Experimental Medicine (IKEM) Prague, scheduled for regular surveillance biopsy, and consented to participate in the study. Recipients transplanted for HCV-related cirrhosis were excluded unless they achieved sustained virologic response after the antiviral treatment. The study conformed to the ethical guidelines of the 1975 Declaration of Helsinki and was approved by the Joint Ethics Committee of the Institute for Clinical and Experimental Medicine and Thomayer Hospital. All patients signed informed consent. Patients received different immunosuppression protocols based either on cyclosporin or tacrolimus depending on transplantation era. All relevant clinical data are provided in Supplementary Table S1.

### Clinical Assessment

All patients were followed by transplant physicians at the Center at regular intervals. At the time of biopsy (±1 month), clinical assessment (blood pressure, body weight, and abdominal circumference measurement), as well as laboratory evaluations of bilirubin, ALT, AST, GGT, ALP, total cholesterol, LDL- and HDL-cholesterol, and triglycerides were performed. Patients further underwent examination focused on insulin sensitivity: fasting glucose, HbA1c concentration, C-peptide, and fasting insulin. Based on these data, HOMA (homeostatic model assessment) and QUICKI (quantitative insulin sensitivity check) indexes were calculated. All biochemical analyses were performed by the in-house routine biochemistry laboratory according to the approved protocols. Total Bilirubin, ALT, AST, GGT, ALP, total cholesterol, HDL-cholesterol, triglycerides a glucose were determined on automatic Architect c16000 Analyzer (Abbott Diagnostics) using the following kits: Bilirubin Total: 6L45-21, ALT: 8L92-41, AST: 8L91-41, GGT: 7D65-21, ALP: 7D55-22, Cholesterol: 7D62-21, HDL-Cholesterol: 3K33-21, Triglycerides: 7D74-21, and Glucose: 3L82-41). LDL-cholesterol was calculated using the Friedwald equation. HbA1c was measured by HPLC on Tosoh Automated Glycohemoglobin Analyzer HLC 723 G8 (Tosoh Corp., Japan). C-peptide was determined by immunochemistry (ECLIA method) on Cobas e801 Analyzer (Roche Diagnostics). Insulin was measured using an immunoradiometric (IRMA) kit (Beckman Coulter, Prague, CR).

### Histological Assessment

Liver graft biopsies were obtained either by Menghini technique (1.6 G needle) or by true cut biopsy device. Formalin-fixed, paraffin-embedded liver graft tissue was routinely processed and 4 μm thick serial sections were stained according to the standard protocol of our laboratory with hematoxylin and eosin, elastic-van Gieson and Shikata's orcein method, Perls' Prussian Blue reaction, and periodic acid-Schiff technique after diastase digestion. For the purpose of the study, all biopsies were evaluated by two experienced pathologists. All biopsies were classified according to the Kleiner's histological scoring system for NAFLD (23). NAS (NAFLD activity score) was calculated as the sum of the scores for the hepatocellular steatosis (0–3), lobular inflammation (0–3), and ballooning (0–2). Additionally, the extent of the liver graft fibrosis stage was classified as proposed by Kleiner et al. (23), i.e., (stage 1A—mild perisinusoidal fibrosis in zone 3; stage 1B—moderate perisinusoidal fibrosis in zone 3; stage 1C—portal/periportal fibrosis; stage 2—perisinusoidal and portal/periportal fibrosis; stage 3—perisinusoidal and bridging fibrosis; stage 4—cirrhosis). Liver graft steatosis was defined as the presence of liver fat in ≥5% hepatocytes on hematoxylin-eosin staining.

### RNA Isolation, Transcriptomics, qPCR Validation

Liver tissue (20–30 mg) was placed into RNAlater (ThermoFisher Scientific, Waltham, MA, USA) immediately after the biopsy and then stored in −80°C until further processed. Total RNA was isolated using TRIzol Reagent (ThermoFisher Scientific, Waltham, MA, USA) and RNeasy Mini Kit (Hilden, Germany). The quality and integrity of the total RNA were evaluated on the Agilent 2100 Bioanalyzer system (Agilent, Palo Alto, CA, USA). Only samples surpassing the minimal quality threshold (RIN > 8.0) were used in the subsequent transcriptomic assessment. Microarray experiments were performed using the GeneChip™ Human Gene 2.1 ST Array Strip (Thermo Fisher Scientific, Waltham, MA, USA) on Affymetrix GeneAtlas™ system (Thermo Fisher Scientific, Waltham, MA, USA) according to manufacturer's instructions. The quality control of the chips was performed using Affymetrix Expression Console (Thermo Fisher Scientific, Waltham, MA, USA); Partek Genomics Suite 7.0 (Partek Inc., St. Louis, Missouri, USA) was used for subsequent data analysis. Transcriptomic data were then processed by standardized sequence of analyses (hierarchical clustering and principal component analysis, gene ontology, gene set enrichment, “Upstream Regulator Analysis,” “Mechanistic Networks,” “Causal Network Analysis,” and “Downstream Effects Analysis”) using Ingenuity Pathway Analysis (IPA hereafter, Qiagen Redwood City, Inc., Redwood City, CA, USA). For qPCR validation, the expressions of 19 selected transcripts were verified in 47 randomly chosen samples (19 control, 28 steatotic). The complete procedure involving reverse transcription (TATAA GrandScript cDNA Supermix; TATAA Biocenter AB, Göteborg, Sweden), pre-amplification (iQ Supermix, Bio-Rad Laboratories, Inc., Hercules, CA, USA + Applied Biosystems TaqMan Assays; Thermo Fisher Scientific, Waltham, MA, USA) and High-throughput qPCR System BioMark (Fluidigm Corporation, South San Francisco, CA, USA) analysis using 48.48 Gene Expression Dynamic Chip (Fluidigm Corporation, South San Francisco, CA, USA). The primary analysis of the data was performed by Fluidigm Real-Time PCR Analysis 4.1.2, the comparisons were assessed using the Livak analysis method (24) with glyceraldehyde 3-phosphate dehydrogenase (GAPDH) as reference gene (Supplementary Figure S1).

### Statistics

After applying quality filters and data normalization by Robust Multichip Average algorithm, the set of obtained differentially expressed probesets [analysis of variance with hepatocellular steatosis (0 vs. 1, 2, and 3) as fixed effect] was subsequently filtered by false discovery rate (FDR) method implemented in PARTEK Genomics Suite 7.0 (Partek Inc., St. Louis, Missouri, USA). For further analysis, only genes showing statistically significant (FDR < 0.05) differences in expression between patients with vs. without steatosis were included in subsequent analyses. Selected genes and patients were subject to two-dimensional hierarchical cluster analysis with a Pearson correlation coefficient being a measure of dissimilarity. Outcomes of the cluster analysis were displayed in a heatmap. Moreover, selected covariates (time from transplantation, fibrosis, ballooning, inflammation, NAS score) were analyzed with respect to steatosis using a Spearman correlation coefficient. The results were also displayed in heatmaps. Analyses were performed using a statistical package R version 3.4.4 (25). *P*-values < 5% were considered statistically significant.

## Results

### Cohort Comparison

Samples from 91 liver graft recipients (45 men, 46 women) passed all the quality controls and were included in the subsequent analyses. The relevant clinical characteristics are shown in Table 1. The individuals with hepatic steatosis showed higher indices of obesity including waist circumference and BMI. Except for the latter two parameters, the subjects with mild steatosis (grade 1) were comparable in remaining measures with controls. The individuals with more severe steatosis (grade 2+3) in their liver grafts had higher levels of ALT, total cholesterol and triglycerides compared to controls, yet the values of these parameters were still in the normal range. As expected, the parameters associated with insulin sensitivity, i.e., fasting glucose, HbA1c, fasting insulin, C-peptide, and HOMA index were higher in patients with grade 2+3 steatosis compared to controls and for fasting insulin, C-peptide and HOMA index even when compared to subjects with grade 1 steatosis.

**Table 1:** Clinical characteristics of patients without or with graft steatosis.

	**Non-steatosis (*n* = 43)**	**Steatosis grade 1 (*n* = 37)**	**Steatosis grade 2, 3 (*n* = 11)**
Sex (women/men)	25/18	16/21	4/7
Age (years)	51.6 (49.0; 59.7)	61.7^*^ (54.6; 66;6)	58.4 (47.1; 64.5)
Time from Tx (days)	906 (443; 3756)	899 (433; 1922)	751 (482; 1880)
BMI (kg/m^2^)	24.0 (21.5; 28.4)	26.2^*^ (24.4; 31.0)	28.4^*^ (26.8; 32.2)
Waist circumference (cm)	91.0 (17.5; 99.5)	103.0^*^ (89.0; 111.0)	108.0^*^ (102.5; 113.5)
**LIVER FUNCTION TESTS**			
Total bilirubin (μmol/l)	12.1 (9.3; 17.8)	12.5 (9.9; 18.9)	13.5 (8.35; 16.4)
AST (μkat/l)	0.38 (0.32; 0.44)	0.41 (0.33; 0.47)	0.49 (0.38; 0.58)
ALT (μkat/l)	0.4 (0.34; 0.48)	0.45 (0.35; 0.61)	0.59^*^ (0.58; 0.72)
ALP (μkat/l)	1.32 (1.08; 1.16)	1.31 (1.07; 1.69)	1.54 (1.21; 2.18)
GGT (μkat/l)	0.36 (0.28; 0.54)	0.38 0.28; 0.73	0.93^#^ (0.53; 1.44)
**LIPID METABOLISM**			
TAG (mmol/l)	1.0 (0.8; 1.3)	1.1 (0.9; 1.4)	1.8^*^^#^ (1.3; 2.6)
Total cholesterol (mmol/l)	4.5 (3.8; 5.0)	4.4 (3.7; 4.9)	4.8^*^^#^ (4.2; 5.4)
LDL-cholesterol (mmol/l)	2.5 (2,0; 3.0)	2.5 (1.9; 3.0)	3.2 (1.9; 3.4)
HDL-cholesterol (mmol/l)	1.3 (1.1; 1.5)	1.2 (1.0; 1.4)	1.1 (0.8; 1.2)
**GLUCOSE METABOLISM**			
Fasting glucose (mmol/l)	5.2 (4.9; 5.6)	5.2 (4.9; 6.0)	5.8^*^ (5.6; 6.6)
HbA1c (mmol/mol)	37 (32; 40)	37 (32; 43)	41 (36; 47)
C-peptide (nmol/l)	0.7 (0.6; 0.9)	0.8 (0.7; 0.9)	1.2^*^^#^ (0.9; 1.5)
Fasting insulin (μIU/ml)	7.1 (4.35; 8.8)	7.0 (5.2; 9.6)	10.1^*^^#^ (6.9; 18.1)
HOMA-IR	1.554 (0.982; 2.182)	1.647 (1.139; 2.559)	2.646^*^^#^ (1.518; 4.361)
QUICKI	0.337 (0.339; 0.3845)	0.354 (0.332; 0.375)	0.330 (0.308; 0.360)

*Data are given as a median (1st quartile; 3rd quartile). Statistical significance between both groups was calculated by Student's t-test with Bonferroni correction*.

* *p < 0.05 compared to non-steatosis*,

# *p < 0.05 compared do steatosis grade 1. ALT alanine aminotransferase; ALP, alkaline phosphatase; AST, aspartate aminotransferase; BMI, body mass index; CRP, C-reactive protein; GGT, gamma-glutamyl transferase; HDL, high-density lipoprotein; HbA1c, glycated hemoglobin; HOMA-IR, homeostatic model assessment for insulin resistance; LDL, low-density lipoprotein; QUICKI, quantitative insulin sensitivity check index; TAG, triacylglycerol; Tx, transplantation*.

Immunosuppressive medication of patients in both cohorts was comparable (Supplementary Table S2). All participants were treated with calcineurin inhibitor tacrolimus, only a few were receiving cyclosporine. In addition, most of the patients received mycophenolate mofetil, while azathioprine, sirolimus or everolimus were prescribed only to the minor portion of patients. Eleven patients without steatosis and twelve patients with steatosis received antilymphocyte induction with either antithymocyte globulin or anti-CD25 monoclonal antibody (daclizumab or basiliximab). All received a high dose of methylprednisolone during anhepatic phase (immunosuppressive therapy is detailed in Supplementary Table S1). In the subcohorts of patients with steatosis, statin, insulin and PAD treatment was more frequent compared to the patients without steatosis.

In the whole cohort, the prevailing primary diseases leading to liver transplantation were alcoholic cirrhosis and biliary cirrhosis. In patients with graft steatosis, the alcoholic cirrhosis was more frequent (29.2 vs. 11.6% in steatosis group vs. controls, respectively), while in controls, the biliary cirrhosis dominated (18.8 vs. 41.9%). Other diagnoses represented in both groups included hepatitis B (10.4 vs. 2.3% in steatosis group vs. controls, respectively), hepatitis C (all of them genotype 1) (4.2 vs. 7%), autoimmune disorders (8.3 vs. 7%) and cryptogenic cirrhosis (8.3 vs. 2.3%). Less frequent diagnoses distinct from the above comprised 20.8 and 23.3% of steatotic cases and controls, respectively. These diagnoses included polycystic liver disease (*n* = 5), epithelioid hemangioendothelioma (*n* = 3), acute liver failure (*n* = 3), hepatic adenoma (*n* = 2), cholangiocarcinoma (*n* = 2), Wilson's disease (*n* = 2), alpha-1 antitrypsin deficiency (*n* = 1), neuroendocrine carcinoma (*n* = 1), and Rendu-Osler disease (*n* = 1).

### Transcriptome Comparison

In order to find genes significantly associated with steatosis in transplanted liver grafts, we adopted a strategy based on testing the difference of transcriptome profiles between patient's steatotic and non-steatotic cohorts. All subjects with the liver fat content higher than 5% were included in the steatotic group while those with <5% of liver fat were considered as non-steatotic. After correction for multiple testing (FDR < 0.05), we identified 747 significantly differentially expressed transcripts (326 upregulated and 421 downregulated in steatotic samples compared to controls) out of 53,617. The top differentially expressed genes are shown in Table 2, the complete set is provided in Supplementary Table S3.

**Table 2:** Top differentially expressed transcripts.

**Symbol**	**Gene name**	**Fold change**	***p***
*P4HA1*	Prolyl 4-hydroxylase subunit alpha 1	−2.26	1.03E-07
*CYP1A1*	Cytochrome P450 family 1 subfamily A member 1	−1.98	7.75E-05
*IGF1*	Insulin-like growth factor 1	−1.73	3.27E-06
*SHBG*	Sex hormone binding globulin	−1.72	3.10E-07
*SLC2A12*	Solute carrier family 2 member 12	−1.66	2.71E-06
*ENST00000560967*	Predicted non-coding transcript	−1.59	3.46E-07
*IGFBP2*	Insulin-like growth factor binding protein 2	−1.58	7.75E-08
*PROZ*	Protein Z, vitamin K dependent plasma glycoprotein	−1.50	6.29E-04
*APOA4*	Apolipoprotein A4	1.55	1.95E-04
*ME1*	Malic enzyme 1	1.55	4.30E-05
*SERPINE1*	Serpin family E member 1	1.56	5.73E-04
*PLIN1*	Perilipin 1	1.65	5.33E-05
*SCUBE1*	Signal peptide, CUB domain and EGF like domain containing 1	1.66	3.42E-07
*MAMDC4*	Mam domain containing 4	1.67	2.31E-07
*DOPEY2*	Dopey family member 2	1.69	3.43E-04
*LOC101928635*	Uncharacterized LOC101928635	1.70	2.73E-04

*Only the transcripts showing >50% difference in fold change of gene expression in steatotic vs. non-steatotic grafts ratio are shown. Unadjusted p-values are indicated (FDR < 0.05 cut-off value is 8.29 E-04)*.

**Figure 1:**
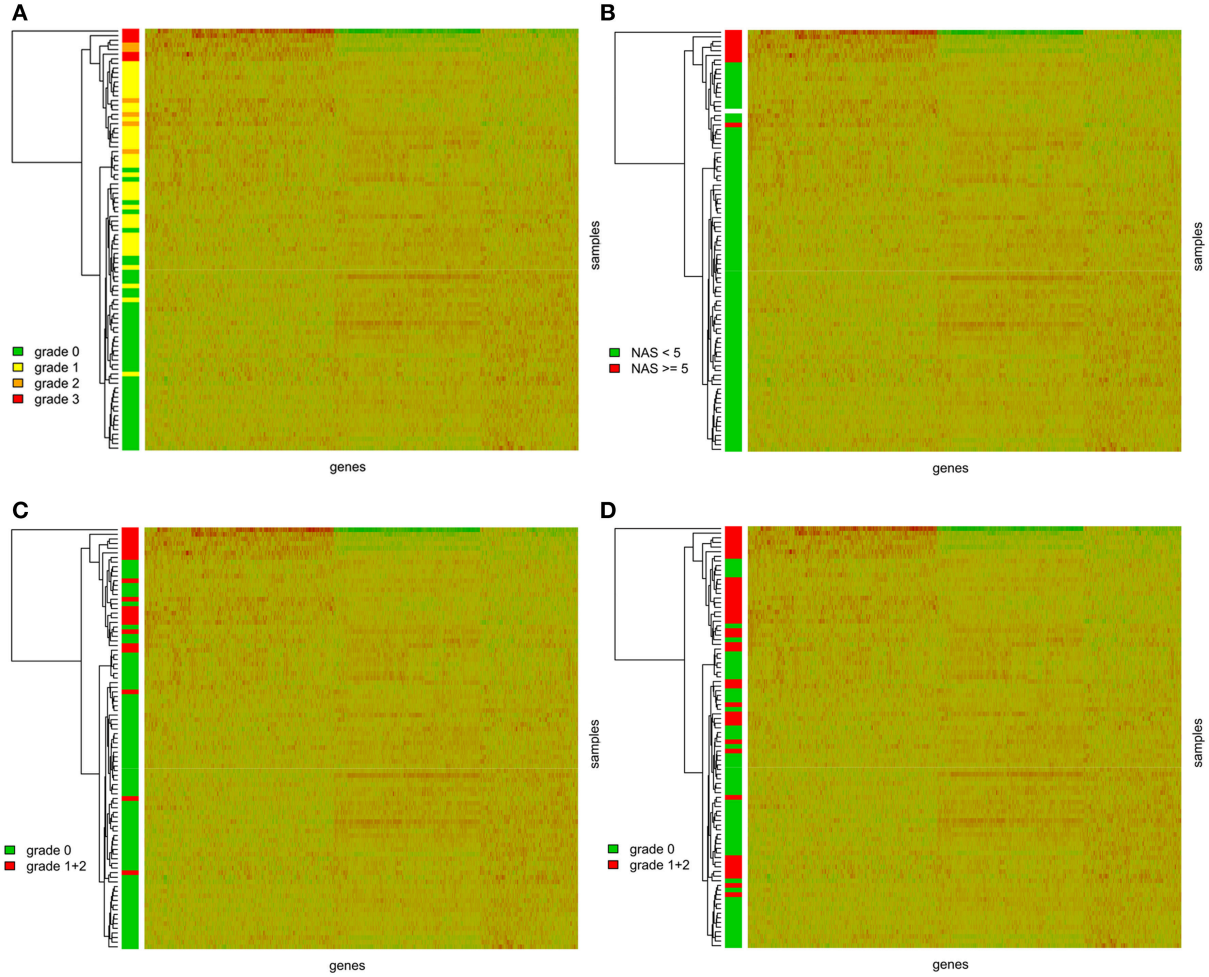
Gene expression heatmaps with the clustering dendrogram of samples. Samples are colored according to **(A)** the grade of steatosis classified according to the Kleiner's histological scoring system for NAFLD (23); **(B)** the NAS score. NAS score was calculated as the sum of the scores for the hepatocellular steatosis (0–3), lobular inflammation (0–3), and ballooning (0–2); **(C)** the ballooning; **(D)** grade of inflammation.

We further performed hierarchical clustering of the genes selected in the previous step. Most of the graft recipients tended to form separate clusters according to the level of steatosis (Figure 1A). This analysis, expectedly, revealed two distinct clusters of transcripts based on their expression patterns. We further wanted to know whether the genes differentially expressed in steatotic grafts show association with other features of liver histology typical for NAFLD/NASH and therefore we displayed inflammation, ballooning, fibrosis level, and NAS score, in the clustering heatmaps as well. We found no association between steatosis and fibrosis grade (*p* = 0.8877) (Supplementary Figure S2) or the time interval from transplantation (*p* = 0.2873) (Supplementary Figure S3) respective. On the other hand, the graft recipients showed significant associations between steatosis and the NAS score (*p* < 0.0001), ballooning (*p* < 0.0001), and inflammation (*p* < 0.0001) (Figures 1B–D).

### Identification of Deregulated Metabolic Pathways

In order to identify the metabolic processes and functions deregulated in steatotic grafts we subjected the set of 747 differentially expressed genes to systematic set of gene enrichment, clustering and network analyses using several dedicated tools and databases—IPA, KEGG (Kyoto Encyclopedia for Genes and Genomes) and DAVID (Database for Annotation, Visualization and Integrated Discovery).

**Figure 2:**
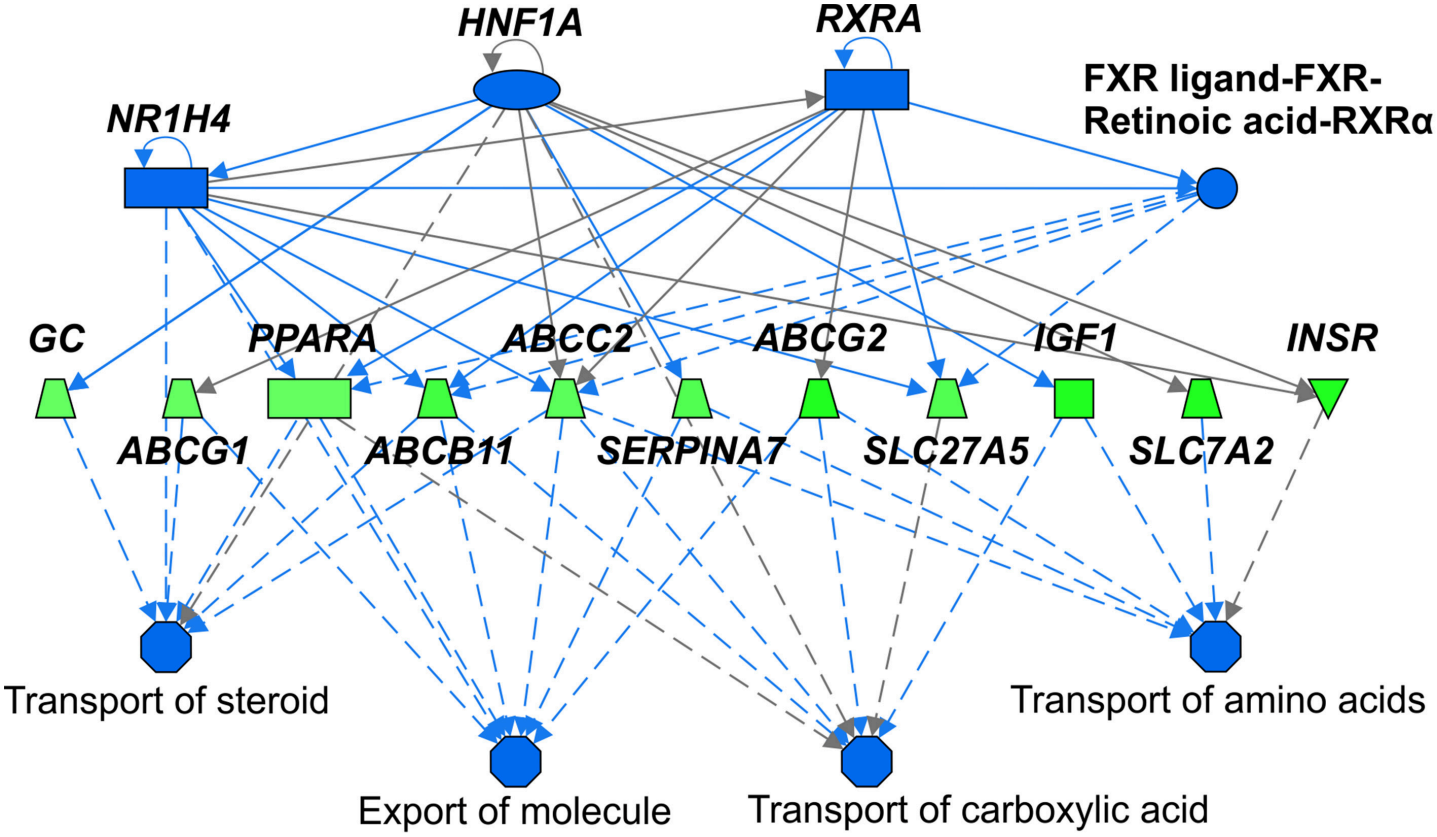
Upstream regulators and downstream processes most affected in steatotic liver grafts. Mechanistic network summarizing main differences in activation (not present) or inhibition (shades of blue) of upstream regulators (top part) and downstream processes (bottom part) in steatotic liver grafts as compared with the non-steatotic transplanted controls. Gene expression comparison between the two groups is shown according to the level of its difference in shades of green (downregulation in steatotic samples) or red (upregulation, not present). Lines depict direct (full lines) or indirect (dashed lines) known interactions. Derivation of the network was performed using Ingenuity Pathways Analysis. The gene symbols are used in accordance with the names approved by the HUGO Gene Nomenclature Committee.

**Figure 3:**
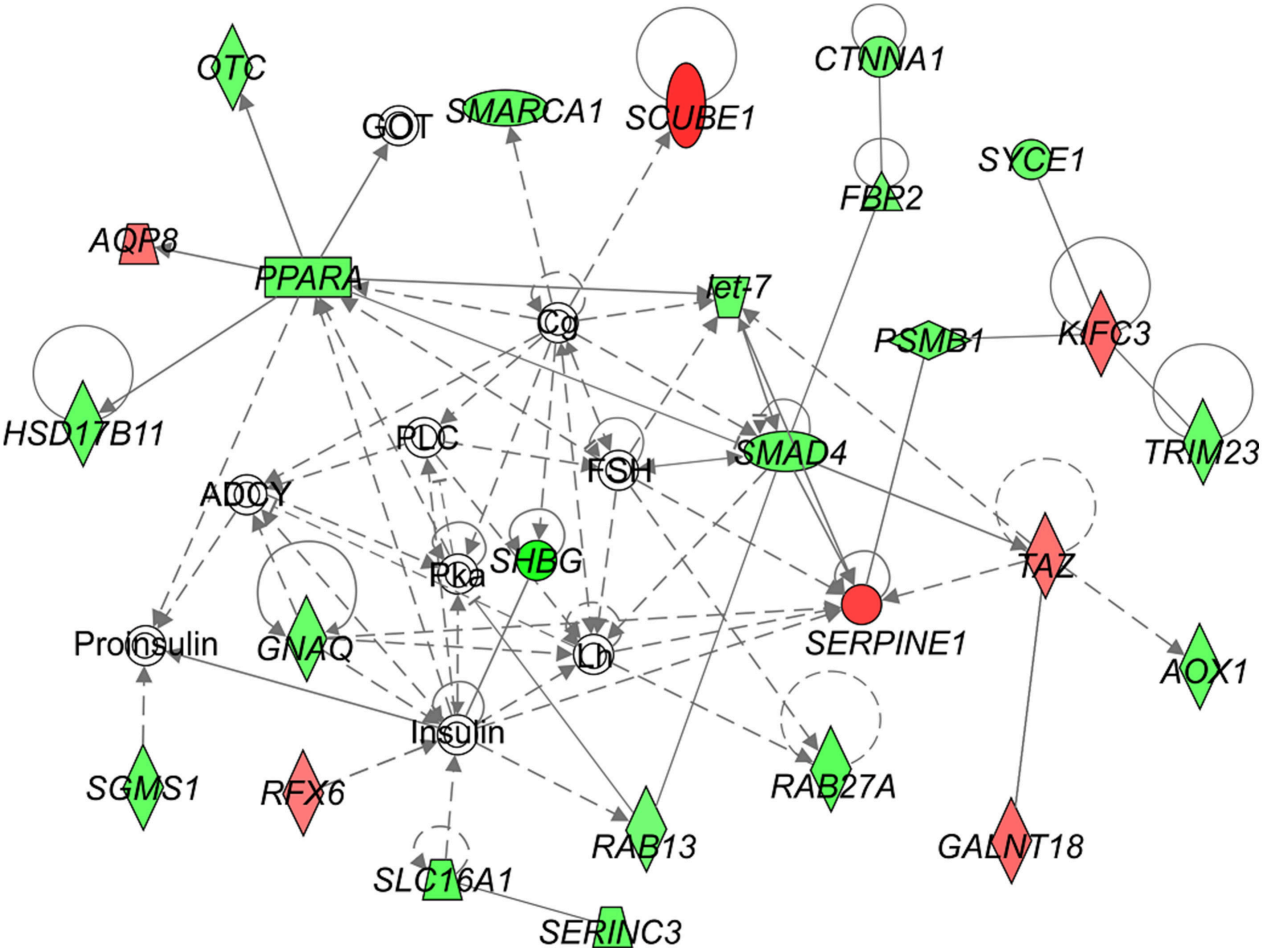
Network analysis of steatosis in transplanted liver grafts. The figure represents the network with the highest score derived using the set of transcripts showing significant difference in expression between steatotic liver grafts and the non-steatotic transplanted controls. The level of difference in gene expression between the two groups is shown in shades of green (downregulation in steatotic samples) or red (upregulation in steatotic samples). Empty symbols indicate entities not found in the experimental dataset of differentially expressed genes. Lines depict direct (full lines) or indirect (dashed lines) known interactions. Derivation of the network was performed using Ingenuity Pathways Analysis. The gene symbols are used in accordance with the names approved by the HUGO Gene Nomenclature Committee.

We identified following significantly enriched biological processes: blood coagulation, bile acid synthesis, and transport, cell redox homeostasis, lipid and cholesterol metabolism, epithelial adherence junction signaling, amino acid metabolism, AMPK and glucagon signaling, transmethylation reactions, and inflammation-related pathways. The list of all significantly deregulated pathways and involved genes is shown in Table 3. Employing IPA, we predicted the potential upstream regulators that may modulate the gene expression in steatotic grafts, including *FXR-ligand-FXR-retinoic acid-RXRa, NR1H4, RXRa, HNF1a, HNF4a*, and *INSR*. Furthermore, using the Regulator effect module, we identified the downstream processes most likely to be perturbed in steatotic grafts, including the transport of specific substances and cellular export machinery (Figure 2). Then we proceeded to generate mechanistic networks based on the observed significant expression shifts. The network reaching the highest arbitrary score in IPA is shown in Figure 3. Several distinct major nodes are apparent including *PPARA* downregulated in steatotic grafts and *SERPINE1* upregulated in steatosis compared to controls. These results combined show systematic shifts of gene expression that distinguish liver grafts with vs. those without signs of steatosis development.

**Table 3:** Metabolic pathways deregulated in steatotic liver.

**Pathway ID**	**Database**	**Description**	***p*-value**	**Involved genes**
GO:0002576	DAVID	Platelet degranulation	0.000	*A2M, F5, FGA, IGF1, SERPINE1*
hsa04610	KEGG	Complement and coagulation cascades	0.000	*A2M, CPB2, F5, C2, C9, FGA, KLKB1, MBL2, SERPINC1, SERPINE1*
GO:0007596	DAVID	Blood coagulation	0.000	*GNAQ, CPB2, F5, FGA, RAC1, SERPINC1*
GO:0042730	DAVID	Fibrinolysis	0.008	*CPB2, FGA, KLKB1, SERPINE1*
	IPA	Coagulation system	0.008	*A2M, F5, FGA, KLKB1, SERPINC1, SERPINE1*
	IPA	Intrinsic prothrombin activation pathway	0.045	*COL5A3, F5, FGA, KLKB1, SERPINC1*
GO:0006699	DAVID	Bile acid biosynthetic process	0.000	*ABCB11, ACOX2, CYP27A1, CYP7B1, SLC27A5*
hsa00120	KEGG	Primary bile acid biosynthesis	0.030	*ACOX2, CYP27A1, CYP7B1, SLC27A5*
hsa04976	KEGG	Bile secretion	0.010	*ABCC2, ATP1B1, SLC27A5, ABCG2, AQP8, ABCB11, ATP1A1*
hsa02010	KEGG	ABC transporters	0.042	*ABCA6, ABCB11, ABCC2, ABCG1, ABCG2*
GO:0045454	DAVID	Cell redox homeostasis	0.022	*ADI1, ALDH1A1, ALDH6A1, AOX1, CBS, CYP1A1, CYP27A1, CYP7B1, LIPF, ME1, MTHFD1, MAOB, NOS1, P4HA1, SESN3*
hsa01230	KEGG	Biosynthesis of amino acids	0.003	*ACO1, ASS1, CTH, CBS, MAT1A, MAT2B, OTC, PSAT1*
	IPA	Cysteine biosynthesis/homocysteine degradation	0.027	*CBS/ CBSL, CTH*
	IPA	S-adenosyl-L-methionine biosynthesis	0.045	*MAT1A, MAT2B*
hsa03320	KEGG	PPAR signaling pathway	0.036	*ACOX2, CYP27A1, PLIN1, PPARα, PCK2, SLC27A5*
	IPA	FXR/RXR activation	0.000	*ABCB11, ABCC2, AKT1, APOA4, APOF, C9, CYP27A1, FETUB, FGA, GC, ITIH4, ORM1, PCK2, PON3, PPARα, PPARGC1α, SLC27A5*
	IPA	LXR/RXR activation	0.013	*ABCG1, APOA4, APOF, C9, FGA, GC, IL18RAP, ITIH4, ORM1, PON3*
	IPA	AMPK signaling	0.045	*AKT1, EP300, INSR, KAT2B, MAPK13, PCK2, PIK3R4, PPARGC1α, PPM1D, RAB11A, RAB27A, SIRT1*
hsa04922	KEGG	Glucagon signaling pathway	0.004	*AKT1, EP300, GNAQ, CAMK2D, ITPR2, PPARα, PCK2, PPP4R3B, SIRT1*
	IPA	LPS/IL-1 mediated inhibition of RXR function	0.013	*ABCB11, ABCC2, ABCG1, ACOX2, ALDH1A1, ALDH6A1, IL18RAP, MAOB, MGST2, PPARα, PPARGC1α, SLC27A5, SULT1A2, SULT1C3*
	IPA	Acute phase response signaling	0.036	*A2M, AKT1, C2, C9, FGA, ITIH4, KLKB1, MAPK13, MBL2, ORM1, SERPINE1*
	IPA	Epithelial adherence junction signaling	0.041	*ACVRC1, AKT1, APC, ARPC1A, CDH2, CTNNA1, MET, PARD2, RAC1, RAP1A*

## Discussion

In this study, we identified a set of 747 genes associated with the development of graft steatosis in patients after liver transplantation. Previously, Ryaboshapkina (26) published systematic meta-analysis of available human gene expression studies on liver biopsies and bariatric surgery samples from NAFLD patients. Using regression models, they identified a set of 280 genes showing a consistent association with NAFLD in at least three independent studies. Thirty genes from this list overlap with those identified by us including genes involved in cellular stress (annexins, *DNAJC12*), mitochondrial metabolism (*PPARGC1, ATP1A1*), lipid and cholesterol metabolism (*PPARA, LPIN1*, ABC transporters, *APOF*), regulation of gene expression (*H2AFY*) or extracellular matrix metabolism (*LAMA 2,3/LAMA5, PCOLCE2*). The possible explanation for the difference between our and others' studies may arise from several reasons. First, the cohort represents a rather unique subset in the “garden variety” NAFLD patients given the fact that our study was performed on liver transplant recipients. Therefore, while presumably there are common mechanisms underlying most NAFLD cases, specific changes may be at play in its pathogenesis in the transplanted graft. Also, we cannot exclude the effect of administered immunosuppressive and concomitant therapy (ursodeoxycholic acid, statins). Second, all of the above-mentioned studies were focused on markers of NAFLD progression toward NASH and fibrosis; however, an only minor fraction of our patients exhibited these pathologies.

Traditionally NAFLD is one of the hallmarks of insulin resistance. In our cohort of patients with graft steatosis, we saw clear markers of deregulated glucose metabolism in individuals with steatosis grade 2+3 but not in those with the only mild form of the disease (grade 1). Because of the limited cohort size, we had to combine all steatotic samples into one set in order to perform a transcriptomic analysis. When we performed hierarchical clustering on the selected set of NAFLD-associated genes we observed clear grouping of individuals with grade 3 steatosis but subjects with grade 2 steatosis did not form a separate cluster and were mixed with both grade 1 and grade 3 samples in a diffused manner. This result indicates that the expression of NAFLD-associated genes is altered even in the early stages of NAFLD prior to the onset of insulin resistance.

Within our subset of deregulated genes, we identified metabolic pathways associated predominantly with cholesterol and bile acid metabolism, inflammation, lipid metabolism, blood coagulation, and oxidative stress. Interestingly, similar pathways were unveiled by meta-analysis performed by Wruck et al. (27) on NAFLD datasets published by Ahrens (28), du Plessis (29), Horvath (30), and Wruck (31). Several deregulated pathways are associated with cholesterol metabolism. There is a growing body of evidence that poor NAFLD outcome, i.e., progression toward NASH and cirrhosis, is not associated with triglyceride accumulation *per se* but rather with altered cholesterol homeostasis and free cholesterol accumulation (32). In our cohort of patients, several pathways profoundly involved in cholesterol metabolism (FXR/RXR activation, LXR/RXR activation, bile acid biosynthesis, bile acid excretion, ABC transporters) were significantly downregulated in steatotic grafts. Consequently, this implicates that cholesterol conversion to bile acids, cholesterol efflux to the bile as well as cholesterol transport to apo-A1 and HDL-C formation were reduced.

Farnesoid X receptor (FXR) pathway downregulation in grafts that developed steatosis corroborates the data on this major bile acid sensor and metabolism regulator (33) involved in the gut-liver axis homeostasis. Observations showing that activation of FXR directly leads to decrease in liver lipogenesis and amelioration of insulin sensitivity served as the rationale for the development of FXR agonists (e.g., obeticholic acid) as potential therapeutic agents for NAFLD (34, 35). Taken together, all these data suggest that alteration of cholesterol homeostasis, cholesterol accumulation within hepatocytes, and down-regulation of bile acid synthesis are characteristic features of graft steatosis and may play a role in NAFLD progression.

As expected, we identified deregulation of lipid metabolism-related pathways, i.e., the down-regulation of PPAR signaling and AMPK signaling. This metabolic milieu setting promotes the triglyceride accumulation and weakens their oxidation what further establish the pro-steatotic feedback loop. Our previous study demonstrated that high serum triglyceride level was an independent risk for graft steatosis (17).

Oxidative stress (36) and inflammation (37) belong to the well-recognized components of NAFLD/NASH pathophysiology. In our cohort of patients, we observed significant down-regulation of 15 genes involved in the maintenance of redox homeostasis what implicates at least an increased susceptibility to the oxidative stress. The patients in our cohort did not manifest histological markers of severe inflammation and therefore it is not surprising that we did not find an increased activation of pro-inflammatory cytokines and other related genes that were reported in transcriptomic studies focused on NAFLD progression toward NASH or fibrosis (26, 38). Furthermore, all liver graft recipients are subjected to immunosuppressive treatment. Nevertheless, we detected the up-regulation of LPS/IL-1 mediated inhibition of RXR function suggesting the presence of subclinical inflammation or infection. RXR inactivation results in attenuation of the expression of hepatic transport and biosynthesis enzymes (ABC, CYP) what, together with other factors, may contribute to the described metabolic alterations in the liver.

We observed down-regulation of S-adenosyl-L-methionine biosynthetic pathway, particularly S-adenosylmethionine synthetases *MAT1A* and *MAT2B*, in steatotic grafts. Their product S-adenosylmethionine is a cofactor involved in methyl group transfers. It is essential for numerous biological processes like methylation of phospholipids that affects membrane fluidity (39), or epigenetic silencing via methylation of gene promoter regions (40). Its role in NAFLD/NASH development was unraveled in experimental animal models (41) and confirmed in human studies (42–44). Importantly, *MAT1A* gene expression in the liver can distinguish between patients with mild vs. advanced NAFLD (38, 45). In our cohort with rather mild steatosis, we found modest but significant downregulation of *MAT1A* and *MAT2B*. Together with other findings, our data indicate that the expression of these genes may serve as one of the indicators of NAFLD progression.

In our study, several pathways associated with blood coagulation (intrinsic prothrombin pathway, platelet degranulation, fibrinolysis and complement/coagulation cascade) were significantly deregulated. Rather surprisingly, their activation pattern was incoherent. In accordance with previously published observations that consider NAFLD to be the pro-thrombotic state (46), we confirmed significantly increased expression of *SERPINE1* (*PAI-1*). In contrast, all other genes involved in blood coagulation pathways were down-regulated. We could speculate that this downregulation may either represent a counter-regulatory mechanism compensating for the increased risk of thrombosis or reflect the decreased general proteosynthesis in the transplanted liver.

In an attempt to identify the major drivers of the observed gene expression changes, we identified *NR1H4, HNF1*α*, HNF4*α*, RXR*α, and *FXR-RXR* among top upstream regulators that govern the expression of the number of downstream targets differentially expressed in steatotic grafts. These transcription factors belong to the family of liver-enriched transcription factors that regulate hepatocyte-specific gene transcription (47). They control numerous functions including glucose and fatty acid metabolism, synthesis of blood coagulation factors, detoxification (CYP450 activity) and biliary metabolism. In our cohort of patients, we did not observe the downregulation of HNF transcription factors itself but we observed deregulation of many of their downstream targets. However, the activity of HNF transcription factors is regulated on several posttranscriptional levels and our findings suggest that deregulation of these transcription factors may represent an early event in the deterioration of overall hepatic function.

Not surprisingly, hierarchical clustering analysis confirmed the association between the selected set of differentially expressed genes and groups of graft recipients based on the level of steatosis. More interestingly, we found that based on the expression patterns of these “pro-steatotic” genes the graft recipients tend to cluster also according to the other histological markers of NAFLD activity score (NAS), inflammation or ballooning (a histological marker of mitochondrial dysfunction). This finding supports the hypothesis that our set of genes may be indicative for the negative prognosis of further NAFLD development in the transplanted liver.

We have found no association between gene expression pattern and graft fibrosis, despite fibrosis is considered an important feature of NAFLD progression. The explanation could be the low number of patients with high NAS score (with advanced NAFLD), which comprised <10% in our study with extensive follow up of our recipients (17). Other reason for this finding could arise from the nature of population studied, where many other factors besides NAFLD could contribute to graft fibrosis, namely subclinical graft rejection, disease recurrence, and transfer from the donor, which could not be excluded. Importantly we have found no association of gene expression pattern and time elapsed between engraftment and liver biopsy.

Despite its merits, our study has limitations that are important to acknowledge. First, prior further considerations, validation of the ascertained expression profiles and networks must be performed on an independent cohort. Second, the cross-sectional design of the study precludes the use of discrete data points to reflect NAFLD development. The fact that a particular gene or pathway is differentially expressed in NAFLD patients may identify it as a marker but it does not imply its causative role in NAFLD development. Further mechanistic studies are necessary to verify or disprove the role of identified genes in the disease progression.

## Conclusions

In summary, we present transcriptomic profile and pathway deregulation patterns distinguishing steatosis-prone from steatosis-free transplanted liver grafts. While some parts of the identified molecular signature are shared with those found in NAFLD in non-transplanted individuals, the unique revealed characteristics may, upon validation in independent studies, lay the groundwork for the establishment of predictive assessment of NAFLD risk in liver grafts.

## Ethics Statement

The study conformed to the ethical guidelines of the 1975 Declaration of Helsinki and was approved by the Joint Ethics Committee of the Institute for Clinical and Experimental Medicine and Thomayer Hospital. All patients signed informed consent. Patients received different immunosuppression protocols based either on cyclosporin or tacrolimus depending on transplantation era. All relevant clinical data are provided in Supplementary Table S1.

## Author Contributions

OŠ, MC, and PT designed the study, analyzed the data, discussed the manuscript, coordinated and directed the project, developed the hypothesis, and wrote the manuscript. IM, DE, and PT recruited the patients. HD, MH, MB, NĎ, and BC performed the analyses. VČ and LŠ analyzed the data and performed the statistical evaluation. All authors read and approved the final manuscript and joined the analysis and interpretation of data.

### Conflict of Interest Statement

The authors declare that the research was conducted in the absence of any commercial or financial relationships that could be construed as a potential conflict of interest.
