# Supplementary material for: Hepatic Gene Expression Profiles Differentiate Steatotic and Non-steatotic Grafts in Liver Transplant Recipients
Source: Front Endocrinol (Lausanne). 2019 Apr 30;10:270. doi: 10.3389/fendo.2019.00270 (PMC6502969; doi:10.3389/fendo.2019.00270)
Supplement: Supplementary file 2 [file Table_2.DOCX]

**Supplementary table S2.** Medication of patients.

|  | non-steatosis | | steatosis grade 1 | | steatosis grade 2,3 | |
| --- | --- | --- | --- | --- | --- | --- |
|  | n | % | n | % | n | % |
| tacrolimus | 37 | 86 | 33 | 89 | 11 | 100 |
| cyclosporine | 6 | 14 | 3 | 8 | 0 | 0 |
| MMF | 27 | 63 | 23 | 62 | 7 | 64 |
| MMF + sirolimus | 0 | 0 | 1 | 3 | 0 | 0 |
| AZA | 1 | 2 | 1 | 3 | 0 | 0 |
| sirolimus | 1 | 2 | 2 | 5 | 0 | 0 |
| everolimus | 0 | 0 | 1 | 3 | 0 | 0 |
| corticoids | 22 | 51 | 16 | 43 | 4 | 36 |
| ATG | 7 | 16 | 3 | 8 | 0 | 0 |
| anti-CD25 mAb | 4 | 9 | 4 | 11 | 5 | 45 |
| insulin | 4 | 9 | 8 | 22 | 3 | 27 |
| PAD | 2 | 5 | 2 | 5 | 1 | 9 |
| statins | 5 | 12 | 7 | 19 | 3 | 27 |

MMF mycophenolate mofetil; AZA azathioprine; PAD other antidiabetics; ATG antithymocyte globulin; anti-CD25 mAb anti-CD25 monoclonal antibody.
