# Supplementary material for: Hepatic Gene Expression Profiles Differentiate Steatotic and Non-steatotic Grafts in Liver Transplant Recipients
Source: Front Endocrinol (Lausanne). 2019 Apr 30;10:270. doi: 10.3389/fendo.2019.00270 (PMC6502969; doi:10.3389/fendo.2019.00270)
Supplement: Supplementary file 4 [file Image_1.pdf]

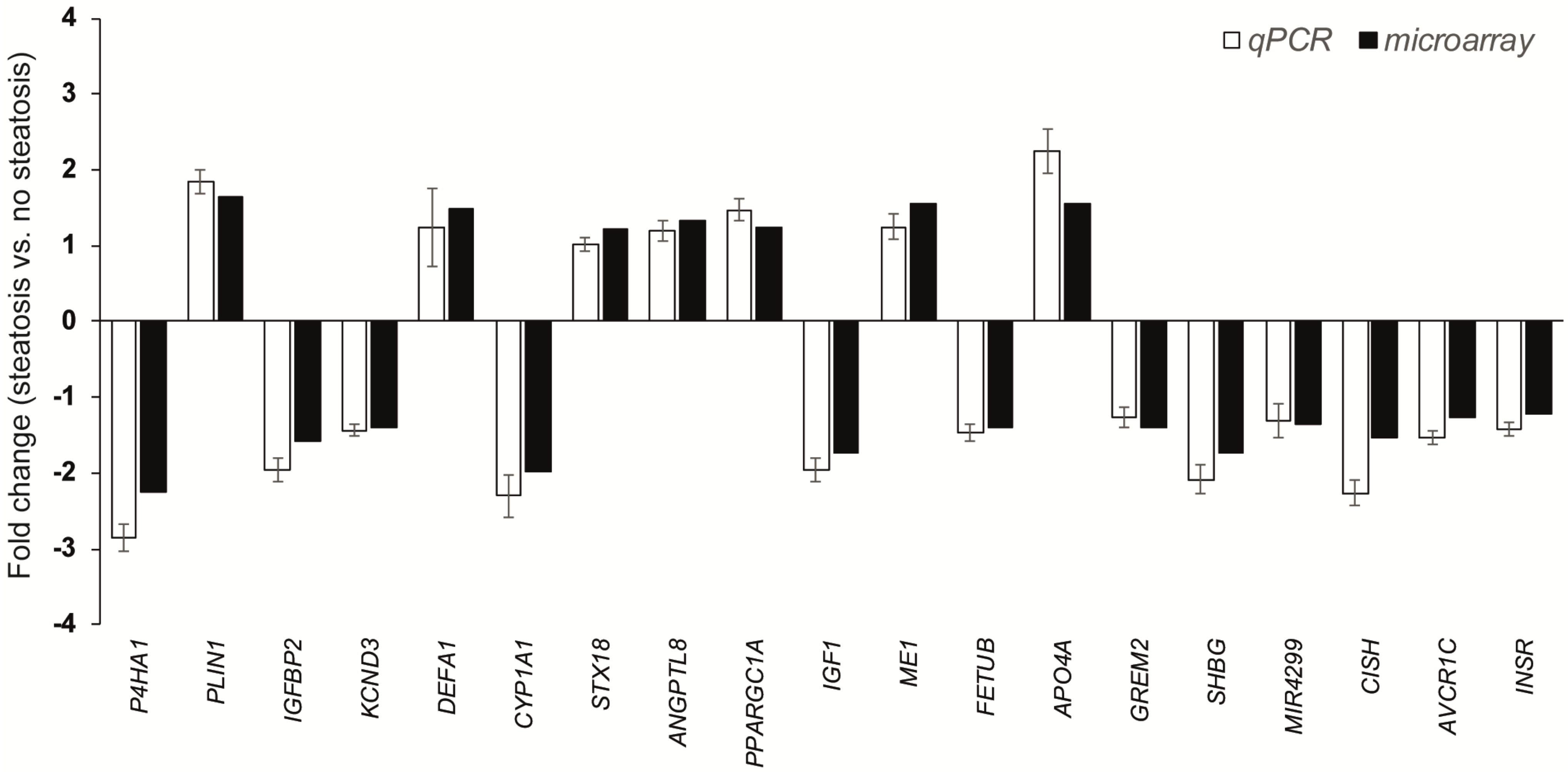

Supplementary Figure S1: Validation of transcriptomic results by qPCR. Fold changes are indicated for comparison of expression between steatotic vs. non-steatotic liver graft samples (qPCR: white bars; microarray: black bars). The microarray fold-change values are shown based on Partek Genomics Suite analysis (see Methods), the qPCR results are expressed as the mean fold-change + standard error of mean for each transcript. The gene symbols are used in accordance with the names approved by the HUGO Gene Nomenclature Committee.
