# Supplementary material for: Hepatic Gene Expression Profiles Differentiate Steatotic and Non-steatotic Grafts in Liver Transplant Recipients
Source: Front Endocrinol (Lausanne). 2019 Apr 30;10:270. doi: 10.3389/fendo.2019.00270 (PMC6502969; doi:10.3389/fendo.2019.00270)
Supplement: Supplementary file 5 [file Image_2.pdf]

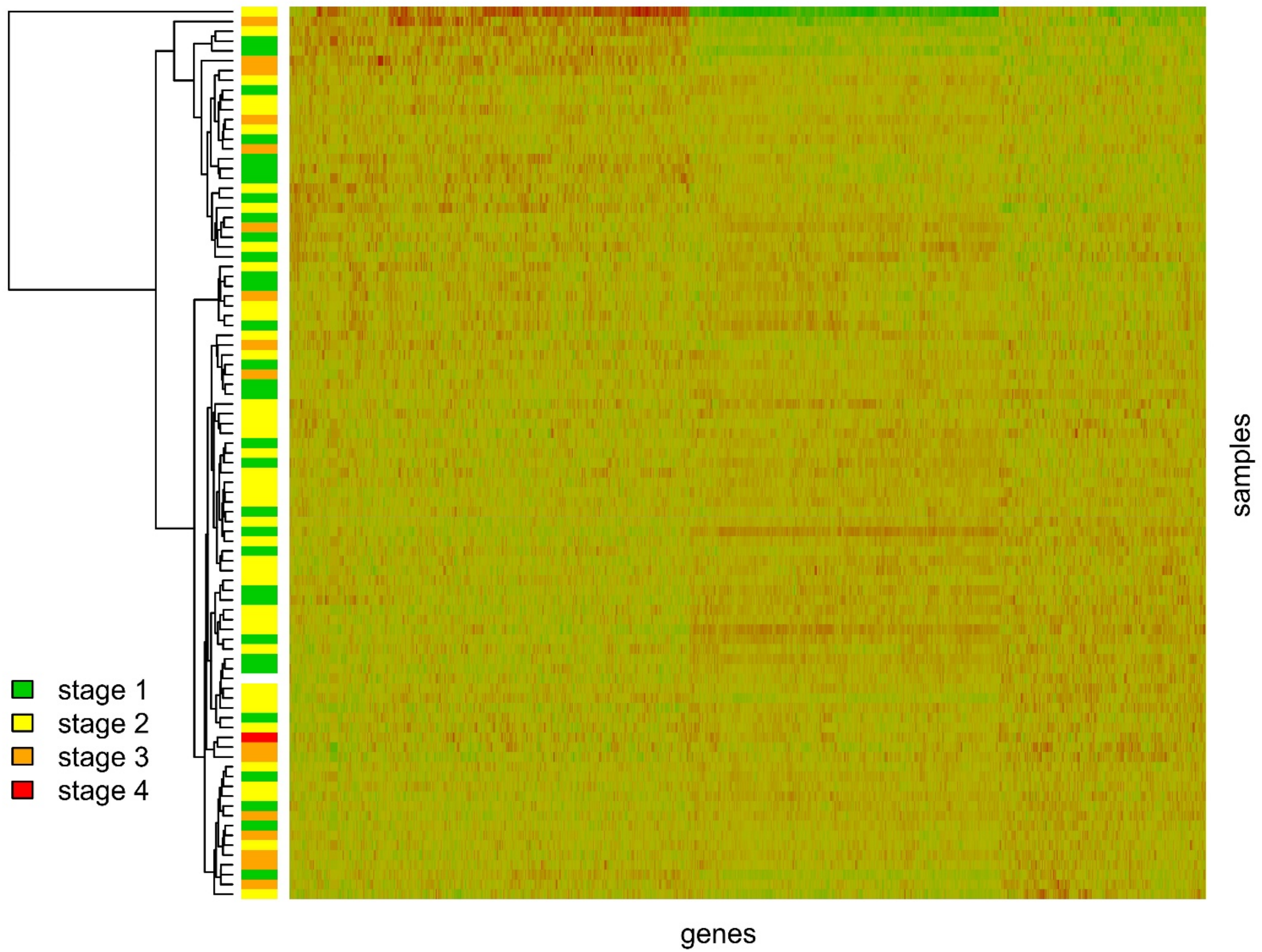

Supplementary Figure S2: *Gene expression heatmap with the clustering dendrogram of samples*. Samples are colored according to the fibrosis stage. The extent of the liver graft fibrosis stage was classified as proposed by Kleiner et al [19], i.e. (stage 1A - mild perisinusoidal fibrosis in zone 3; stage 1B - moderate perisinusoidal fibrosis in zone 3; stage 1C - portal/periportal fibrosis; stage 2 – perisinusoidal and portal/periportal fibrosis; stage 3 – perisinusoidal and bridging fibrosis; stage 4 – cirrhosis).
