# Supplementary material for: Hepatic Gene Expression Profiles Differentiate Steatotic and Non-steatotic Grafts in Liver Transplant Recipients
Source: Front Endocrinol (Lausanne). 2019 Apr 30;10:270. doi: 10.3389/fendo.2019.00270 (PMC6502969; doi:10.3389/fendo.2019.00270)
Supplement: Supplementary file 6 [file Image_3.pdf]

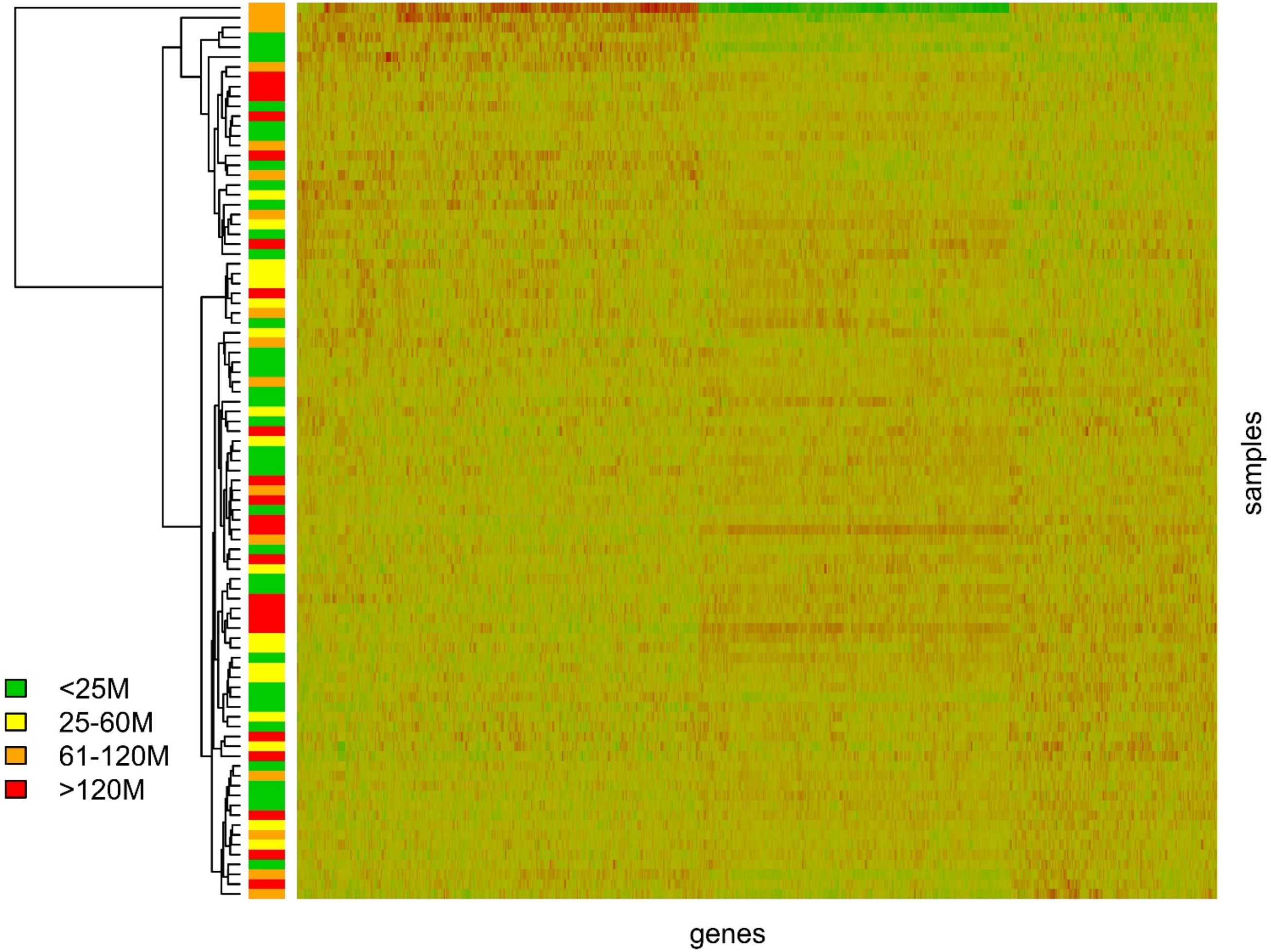

Supplementary Figure S3: *Gene expression heatmap with the clustering dendrogram of samples*. Samples are colored according to the time from transplantation. The time interval is given in months (M).
